# Intimate Partner Violence among Pregnant Women and Postpartum Depression in Vietnam: A Longitudinal Study

**DOI:** 10.1155/2019/4717485

**Published:** 2019-04-24

**Authors:** Tran Tho Nhi, Nguyen Thi Thuy Hanh, Nguyen Duc Hinh, Ngo Van Toan, Tine Gammeltoft, Vibeke Rasch, Dan W. Meyrowitsch

**Affiliations:** ^1^Institute for Preventive Medicine and Public Health, Hanoi Medical University, No.1 Ton That Tung Street, Khuong Thuong, Dong Da, Hanoi, Vietnam; ^2^Department of Anthropology, University of Copenhagen, Øster Farimagsgade 5, DK-1353 Copenhagen K, Denmark; ^3^Department of Obstetrics and Gynecology, Odense University Hospital, 5000 Odense C, Denmark; ^4^Department of Clinical Research, University of Southern Denmark, 5230 Odense M, Denmark; ^5^Department of Public Health, Faculty of Health Sciences, University of Copenhagen, Øster Farimagsgade 5, DK-1014 Copenhagen, Denmark

## Abstract

**Background:**

Exposure to intimate partner violence during pregnancy is associated with a wide range of adverse reproductive health outcomes. However, detailed knowledge on the association between specific types of exposure to partner violence and postpartum depression is limited.

**Purpose:**

The aim of the present study was to investigate the association between exposure to emotional violence, physical violence, and sexual violence during pregnancy and postpartum depression among women in northern Vietnam.

**Methods:**

The study was designed as a longitudinal study, which included a total of 1,337 women. The study participants were recruited from 24 communes in Dong Anh District, Hanoi, Vietnam, and interviewed four times: (a) at enrolment (which took place no later than week 24 of the pregnancy); (b) at a gestational age of 30-34 weeks; (c) at delivery; and d) 4-12 weeks after delivery. Emotional, physical, and sexual violence exerted by the intimate partner were measured using a modified version of the questionnaire initially developed by the World Health Organization, and signs of depression were measured by the Edinburgh Postpartum Depression Scale.

**Results:**

More than one-third of the women (35.3%) experienced at least one type of violence during their pregnancy and 8.2% of the women reported postpartum depression. The results of multivariate analyses showed that both physical and sexual violence were statistically significantly associated with postpartum depression (AOR=2.75, 95%CI: 1.19-6.35 and AOR=1.93, 95%CI: 1.01-3.73, respectively).

**Conclusions:**

The results showed strong and statistically significant associations between partner violence and postpartum depression. These findings clearly demonstrate a crucial need for relevant health professionals to identify women who are exposed to partner violence and screen for postpartum depression in order to mitigate the negative mental health outcomes among Vietnamese women.

## 1. Introduction

Depression is a serious mental disorder that significantly affects women of childbearing age [1]. It is predicted that, by the year 2020, it will be the second leading cause of disease burden, globally [2]. Maternal postpartum depression rates fluctuate between 4.3% and 43.9% [3]. The literature reports some risk factors related to postpartum depression (PPD), such as history of depression, low education level, low income, inoccupation, lack of social support, stress, and intimate partner violence (IPV) [4]. IPV directed towards women is associated with a vast diversity of adverse health outcomes, including poor physical and mental health [5, 6]. According to a study conducted by WHO from countries with different social and cultural backgrounds on mental health along with domestic violence against women, a significant percentage of women underwent at least one type of emotional violence (EV) exerted by their intimate partner in the prior 12 months at the time of the study [7], and 30.0% of the women experienced physical and/or sexual violence during their lifetime [6]. During pregnancy, the prevalence of women that are exposed to IPV in Cambodia and Philippines was approximately 2%, while the prevalence in Uganda was 13.5% [8]. In Vietnam, the prevalence of physical violence during pregnancy was 4.7% [9].

Results from recent studies suggest that IPV is strongly associated with PPD [10, 11]. However, most studies on the association between partner violence and pregnancy related outcomes rely on cross-sectional study designs, and there is a general lack of longitudinal studies with a detailed focus on specific types of partner violence as predictors for development of depression after delivery [12]. In Vietnam, several studies on the burden of postdelivery related depression have been carried out by use of cross-sectional study designs [13, 14]. There is, however, only very limited detailed knowledge on the association between partner violence during pregnancy and PPD among Vietnamese women. Furthermore, early identification of women who are exposed to partner violence and are at risk of developing pregnancy related depression will provide the rationale for appropriate interventions aiming at reducing risks of long-term mental illness [15]. The aim of the present study is to analyze the association between emotional, physical, and sexual violence during pregnancy and postpartum depression.

## 2. Methods

### 2.1. Study Setting

The study was conducted in Dong Anh district, Hanoi, Vietnam. The district is located 20 kilometers east of Hanoi city and covers an area of 182.3 km2. The district has a total population of approximately 390,000 people, is divided into 23 communes, and has one town. An estimated 11,600 women seek antenatal care and delivery, annually in two major hospitals in the district (Dong Anh Hospital and Bac Thang Long Hospital).

### 2.2. Study Design, Sampling, and Recruitment

This is a secondary analysis of data from prospective cohort study which followed women during their pregnancy and after delivery during the period from May 1, 2014, to August 30, 2015.

A total of 1,337 pregnant women were invited at baseline to participate in the study, and 1,274 women completed all interviews and were included in the final sample.

Dong Anh Hospital and Bac Thang Long Hospital were chosen to implement this research. Patients from Antenatal Care Clinics (ANCs) and Commune Health Stations (CHS) under the umbrella of these hospitals were invited to answer the questionnaire. Based on monthly report about the number of pregnant women with general information about their name, place of birth, residential address, and estimated gestational age from Population Center, the researchers employed these reports as a sampling frame to collect interviewee for the present study. An information sheet was given to self-reported women with gestational age below 24 weeks to inform them about overall view of the study before they made a final decision. Then, if they volunteered for participation in the study, they would be requested to sign in a consent form. In the next step, a Medical Doctor (research team member) examined ultrasound scanning for all agreeing pregnant women with the aim of estimating accurate gestation age. The first interview was held at a separated room in ANC, while the place for the second and the fourth round of interview was proposed by interviewees.

They chose where they would like to confide. In order to create a private and confidential space for the interviews, the woman's intimate partner and other family members were not informed about the purpose of the study and they were not present during the interviews.

**Figure 1:**
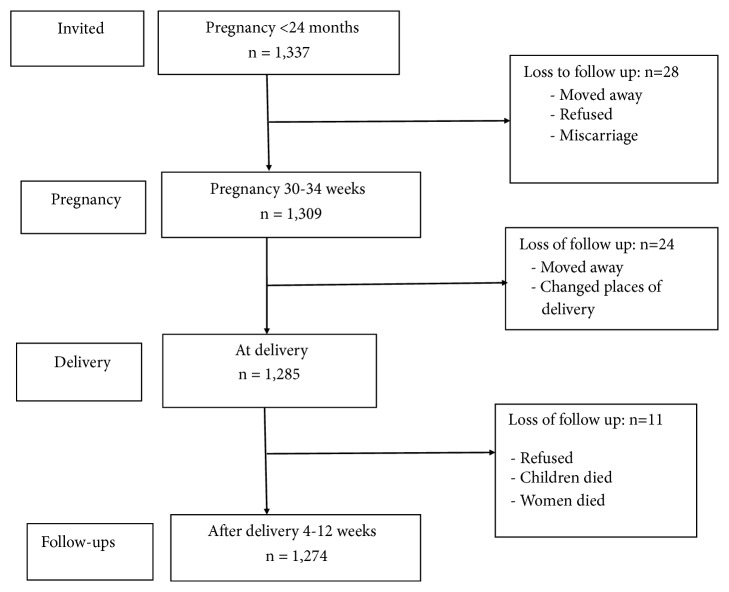
Flow chart of participants.

Data were collected in four surveys. The first one was conducted when participants were at least at 24 weeks' gestation and the second when participants were at a gestational age of 30-34 weeks. After childbirth two assessments of mothers were done at delivery and 4-12 weeks after delivery. The response rate was 95.4%. A total number of 63 women (4.6% of the original sample) were subsequently lost to follow-up due to changes in place of residence/delivery, miscarriage, or refusal. Hence, 1,274 women who participated in all interviews were included in the final analysis (see Figure 1).

### 2.3. Social, Economic, and Health Characteristics

Sociodemographic factors and other possible risks for postpartum depression were collected by study-specific questions. These included the general information regarding the age, educational level, occupation, and place of birth and number of previous pregnancies, mode of delivery, gestational age at birth and the weight of the newborn child, and family support after birth.

### 2.4. Depression

The Edinburgh Postnatal Depression Scale (EPDS) is a widely used 10-item self-reported questionnaire about feelings experienced over the past seven days [16]. The degree of agreement was estimated under the score that ranged from 0 – 3 for each category with four specific short statements. The assessment of level depression from participants was concluded on the total score of EPDS. This tool has been translated into Vietnamese and validated by a study in Vietnam [17]. Previous studies on women in Vietnam found that it has a threshold of 9/10 for routine use in detecting clinically significant depressive symptoms at primary healthcare level [18, 19].

Cronbach's alpha in this study was 0.76 and average interitem covariance was 0.084 with number of items in the scale being 10.

### 2.5. Experiences of Intimate Partner Violence

The data collection instrument used was the Multicountry Study on Women's Health and Life Experiences Questionnaire developed by the World Health Organization (WHO) for studies within public health with focus on interpersonal violence [7]. The questionnaire was developed for use in different cultures and is considered to be cross-culturally appropriate. Violence occurrence was assessed by types (emotional, physical, and sexual violence). Women answering yes to being exposed to at least one of IPV types were classified as having experienced the relevant type of IPV. Each type of violence was divided into two categories as follows* “moderate violence” *and* “severe violence.” *Women were defined as exposed to* “moderate violence”* when they were slapped, pushed, shoved, or thrown with something by their intimate partner. The other category was concluded if respondents experienced hit that could hurt; were kicked, dragged, or beaten; were choked or threatened by weapon.

### 2.6. Statistical Analysis

The completed questionnaires were assessed by the principal investigator in order to detect and correct errors. Hereafter, the data were double-entered in EPI-DATA 3.1 for quality control. All statistical analyses were performed using STATA version 12 for Windows. Logistic regression models were used to identify the correlates of IPV and PPD. We first conducted univariate analyses with each independent variable. All variables with a p-value<0.2, as well as variables of known clinical importance [20], were included in the multivariate model. We identified potential confounding and interaction by comparing the coefficients from the univariate analysis with those from the multivariate model. We assessed interaction by first creating a list of possible pairs of variables in the main effects model that have some scientific basis to interact with each other. We added each potential interaction term, one at a time, to the model containing the main effects and assessed its significance using the likelihood ratio test. This process revealed no significant interaction. The results are presented as Odds Ratios crude (ORs), Odds Ratios adjusted (AORs), and 95% confidence intervals (95%CI) around the respective ORs and AORs.

### 2.7. Ethics Approval and Consent to Participate

Study documents were approved by IRB of the Hanoi Medical University Committee (decision 137/HMU IRB dated November 29, 2013). Subjects were introduced to the purpose of this study and asked to give written informed consent if they agreed to participate and they signed the informed consent form. Participants could withdraw at any time. Their information remained confidential. We also followed the WHO ethical and safety recommendations for researching domestic violence against women [21]. The participants were interviewed in private rooms or in another place that they selected according to their wish. Interventions for victims of partner violence included various approaches, such as psychological support. Women with signs of depression were provided a referral to mental health experts for counseling and treatment.

## 3. Results

### 3.1. Characteristics of Study Participants

The descriptive characteristics of the study participants are presented in Table 1. The mean age of participants was 26 years (range: 16–46 years). Only a small proportion of the women (6.4%) were in the age group 35 years and above. The vast majority of the women were married and lived with their husband (99.5%); more than half had completed basic education (56.3%); and the majority had a job (73.2%) (see Table 1).

### 3.2. IPV during Pregnancy

The women were frequently exposed to IPV during their pregnancy. Over one-third of the women (35.3%) experienced at least one type of violence during their pregnancy, whereas 26.5% experienced IPV during early pregnancy. Emotional violence was the most common type of violence (32.3%) followed by sexual violence (9.8%) and physical violence (3.5%). Among the women 0.8% reported that they were exposed to at least one type of severe physical violence during pregnancy (see Table 1).

### 3.3. The Association between IPV during Pregnancy and Postpartum Depression

A total of 63 women (5.0%) presented with signs of depression during pregnancy and 104 women (8.2%) presented with postpartum depression. The associations between IPV, covariables, and signs of depression after pregnancy are described in Table 2. The results of bivariate analyses indicated that, for the three types of partner violence, physical violence was the strongest determinant for postpartum depression (OR=5.08; 95%CI: 2.58-10.02), followed by sexual violence and emotional violence (OR=1.92; 95%CI: 1.10-3.35 and OR=1.60; 95%CI: 1.07-2.41, respectively).

After adjustment for age of women, occupation of women, level of education, husband's preference for a specific sex of child, age of women at first pregnancy, mode of delivery, gestational age at delivery, and family support after delivery, the results of the multivariate analysis showed that both physical violence and sexual violence remained statistically significantly associated with development of postpartum depression. Women who experienced sexual violence or physical violence had approximately 2-3 times higher odds of presenting with signs of depression as compared to those who were not exposed to these types of violence (AOR=2.75; 95%CI: 1.19-6.35 and AOR=1.93; 95%CI: 1.01-3.73, respectively). In contrast, emotional violence was not significantly associated with postpartum depression (AOR=1.01; 95%CI: 0.60-1.69).

Besides exposure to specific types of violence, other characteristics were also significantly related to development of signs of depression after pregnancy. For instance, being employed by government, private company, or an organization or being a farmer compared to being engaged in small trade was a strong predictor for development of signs of depression (AOR=4.21; 95%CI: 1.82-9.75 and AOR=2.68; 95%CI: 1.13-6.35, respectively). In addition, decreasing levels of education were significantly associated with increasing risks of postpartum depression. Also, the highest risk of postpartum depression was observed among those women who had completed primary school only as compared to those who had completed a university education (AOR=4.59; 95%CI: 1.04-20.29). Other statistically significant predictors for development of postpartum depression were husband's preference for a son (AOR=1.98; 95%CI: 1.15-3.39), low gestational age at delivery (AOR=2.33; 95%CI: 1.03-5.24), high age of women at first pregnancy (AOR=3.07; 95%CI: 1.55-6.07), and lack of family support after delivery (AOR=3.46; 95%CI: 1.87-6.39) (see Table 2).

## 4. Discussion

To the best of our knowledge, this is the first longitudinal study that has examined the association between IPV during pregnancy and signs of depression among Vietnamese women. This study has demonstrated that over one-third of women experienced IPV during pregnancy and emotional violence was the most common type of violence. Women who were emotionally abused during pregnancy were more likely to develop depression than those who were not exposed to emotional violence.

In our study, the prevalence of IPV during pregnancy was 35.3%. This is comparable to a prevalence of 30.7% reported in a Canadian study on IPV during pregnancy as a risk factor for PPD [10] and higher than what was reported in a study on IPV during pregnancy among Mexican women (11.3%) [22]. Although it is difficult to compare rates across cultures due to the many methodological differences in the studies, our findings are consistent with the findings of previous reports from different cultures.

The most common type of IPV experienced during pregnancy was emotional violence (32.3%) followed by sexual violence (9.8%) and physical violence (3.5%). In various studies, comparative results on the prevalence of various forms of violence are documented [15, 23, 24]. The prevalence of exposure to emotional violence exerted by their husband during pregnancy was 10.9% for Mexican American women [22] and 19.9% for women in South Africa [23]; for physical violence and sexual violence experienced during pregnancy the prevalence was 3.8% [15] and 2.8% [23], respectively. In the present study, emotional violence was more likely to occur as compared to physical violence and sexual violence. Similar findings have been documented by a systematic review on IPV during pregnancy that included studies from 72 countries [24]. In Vietnam, the prevalence of physical violence during pregnancy exerted by an intimate partner was 4.7% [9].

The proportion of women with postpartum depression was 8.2%. This rate was of similar magnitude as compared to findings from a study on postpartum depression among Canadian women [25]. However, our findings are within the range of prevalence reported by a systematic literature review that showed that the prevalence rates of postpartum depression ranged from 4.3% to 43.9% [25]. Another study with a specific focus on the Asian region showed that the prevalence of postpartum depression ranged from 3.5% to 63.3%, of which Malaysia and Pakistan had the lowest and highest prevalence, respectively [26]. The cross-sectional study conducted in twelve randomly selected Commune Health Centers from urban and rural districts of Thua Thien Hue Province, Vietnam, on mother-infant dyads one to six months after birth showed that the proportion of women with postpartum depression was 18.1% [9]. The difference in prevalence of depression after delivery in these studies may be due to several factors including the selection of EPDS cut-off points, which differ among studies and countries, the sample size, and possible selection bias related to site of recruitment.

The results of the present study indicate a strong relationship between IPV and postpartum depression. These findings are generally consistent with previous studies that have assessed the associations between IPV and postpartum depression [26, 27]. Additional evidence on the association between IPV and postpartum depression is documented in a systematic review and meta-analysis study which showed that exposure to IPV increased the risk of postpartum depression by 1.5 to 2.0 times [28]. Also, Bonomi and et al. (2009) indicated that the relative risk of PPD among those who experienced IPV during pregnancy was three times higher than those not exposed to IPV [27].

Our study showed that physical violence and sexual violence were statistically significantly associated with signs of postpartum depression. The study has demonstrated the relationship between physically violent behaviors doubled the risk of poorer mental health of abused women as compared to women who had not reported any IPV. The results from a study in India showed that women who experienced physical violence at home were at increased risk of poor mental health [29]. After adjustment in a multivariate analysis there was no significant association between emotional violence and postpartum depression. Lack of association between emotional violence and depression after delivery may be explained by the cultural maternal context in Vietnam. Women were supported partially or fully during the first month after delivery by family members such as the mother, mother-in-law, other relatives, and husband. The help provided ranged from childcare to cooking and other work. So this may work as a protective factor against depression after delivery [30]. On the other hand, emotional violence is more difficult to measure in a survey and most manifestations are not included in criminal or domestic violence laws in Vietnam [7]. To some extent, women are reluctant to perceive that they are victims of violation. From their prospective, some acts, for instance, glaring or damaging household items, are just results of a “hot temper” characteristic and they decided to forgive these actions according to actual context. However, a considerable recorded experiment of being disapproved, humiliated, demeaned, and underestimated, as well as being verbally abused and threatened with violence, is well noted. In addition, living under extreme control, women tend to reduce their sense of self-esteem and independence. It may imply that acceptance in circumstances of suffering violence is observed in women even though they are victims. In some cases, the researcher team coins the term “normal acts” to describe violence action which women believe are usual activities to maintain a relationship [7].

Besides exposure to specific types of violence, other characteristics were also significantly associated with increased risks of postpartum depression. In fact, women who had a husband who preferred having a son had two times increased odds of PPD. The preference for a son is regarded as a growing challenge in a number of Asian countries, especially in rural areas in China, India, Vietnam, Nepal, and Pakistan [29–31]. In Northern Vietnam, parents usually live with their son and rely on their sons economically when they become old, whereas daughters usually live in their husband's home. Many people therefore assign sons a greater value than daughters. Moreover, many people hold that only sons can continue the family line. So the combination of an enormous cultural pressure to produce sons and restrictive population policies can have severe consequences for women's mental health [32, 33]. Therefore, the sex preference of the child goes beyond the parents' wishes and is partly a result of a more general anticipation shared by supporting family members and the surrounding community. Additionally, a study by Fisher et al. found that there was a significant relationship between sex preference and the presence of common mental disorders among women in their early pregnancy. However, this was seen as an “acute stressor” that was rarely associated with persistent depressive symptomatology [10].

There are several limitations of this study. Firstly, violence and depression are sensitive topics that are likely to make some women not disclose their experiences, leading to underestimation of the prevalence and strength of the association between IPV and depression after delivery. Secondly, the women who experienced IPV were given an address where they could obtain support and counseling when needed. Although we did not measure the effect of this intervention on the connections between depression and IPV, it is important to consider the possibility that this support affected the subsequent results. Also, women are encouraged to seek antenatal care services in CHS but there are no data regarding the proportion and characteristics of women who actually use this service. Therefore, when recruiting study participants from the CHS, it is relevant to consider the potential influence of selection bias as a limitation. Finally, the assessment of the outcome of interest, namely, signs of depression, relied on self-reporting rather than clinical assessment, and this may have led to an under- or overestimation of PPD.

## 5. Conclusions

Even though Vietnam has a strong legislative framework promoting gender equality and prohibiting gender-based violence, intimate partner violence against women remains common. Data also suggest that violence is detrimental to mental health of women. The research also documents close associations between women's access to social support, particularly from their natal family, and their risk of exposure to intimate partner violence. Existing policy recommendations to promote screening of pregnant women for IPV have proven difficult to implement. In order to find new pathways to address this persistent health and rights problem in Vietnam, we therefore recommend the following actions: (1) Introduce screening for maternal depression in antenatal care. This can be attained if health staff is trained to screen women for depression during pregnancy and/or after birth, as a routine component of antenatal and newborn care. (2) Provide healthcare services for women experiencing depression during pregnancy and after birth. At present, hardly any healthcare services are available for Vietnamese women who experience mood problems during their pregnancies or after birth. Mental health counseling for this group of women may therefore provide an alternative pathway to interpersonal support for women who are exposed to partner violence.

## Figures and Tables

**Table 1:** Descriptive characteristics of study participants (N = 1,274).

Characteristics	No. of women	% of total
*Age* (years) *(n=1,274)*		
16-24	573	45.0
25-34	620	48.7
≥ 35	81	6.3

*Place of birth (n=1,272)*		
Present community in Dong Anh District	610	47.9
Another community in Dong Anh District	350	27.5
Another district/Another province or city	312	24.6

*Occupation (n=1,273)*		
Government employee/Private company /Organization	408	32.0
Work in private company	349	27.4
Farmer	166	13.0
Small trade	181	14.2
Unemployed/student/homemaker	169	13.4

*Level of education* ^*1*^ * (n=1,274)*		
Primary school	24	1.9
Secondary school	228	17.9
High school	465	36.5
University/college	557	43.7

*Living arrangement in relation to partner (n=1,273)*		
Married and living together	1,267	99.5
Married but living apart	3	0.2
Living with a man, not married	2	0.2
Having a regular partner (sexual relationship), living apart	1	0.1

*Living arrangement in relation to family of birth* ^*2 *^ *and in-laws (n=1,274)*		
Living without family of birth/in-laws	356	27.9
Living with family of birth	62	4.9
Living with in-laws	856	67.2

*Age of women when pregnant for the first time (years) (n=1,273)*		
<20	258	20.3
20-29	973	76.4
≥30	42	3.3

*Number of pregnancies (n=1,274)*		
1	516	40.5
2	345	27.1
≥3	413	32.4

Living children		
1	490	70.4
2	191	27.4
≥3	15	2.2

*Husband's preference for a specific sex of child (n=1,268)*		
Preference for son	575	45.3
Preference for girl	270	21.3
No preference	423	33.4

*Type of partner violence during pregnancy:*		
*Emotional violence during pregnancy*		
Insulted or made her feel bad about herself	65	5.1
Belittled or humiliated her in front of other people	20	1.6
Did things to scare or intimidate her on purpose?	370	29.0
Threatened to hurt her or someone her care about?	16	1.3
Any emotional violence	411	32.3

*Physical violence during pregnancy*		
*Moderate physical violence:*		
Slapped or thrown something	42	3.3
Pushed or shoved	7	0.6

*Severe physical violence:*	10	0.8
Hit that could hurt	9	0.7
Kicked/dragged or beating	4	0.3
Choked or burnt	2	0.2
Threatened to use or actually used a gun, knife, or other weapon	1	0.1
Any physical violence	45	3.5

*Sexual violence during pregnancy*		
Physically forced to have sexual intercourse	111	8.7
Did not want to have sexual intercourse	18	1.4
Forced you to do something sexual that felt degrading	6	0.5
Any sexual violence	125	9.8

*Type of partner violence during early pregnancy (<24 weeks gestational age)*		
At least one type of IPV	337	26.5
Emotional violence	314	24.7
Physical violence	40	3.1
Sexual violence	63	4.9

*At least one type of IPV during pregnancy* ^*3*^	*450*	*35.3 *

^1^Level of education was grouped into primary school (up to grade 5 years), secondary school (grade 6-9 years), high school (grade 10-12 years ) and higher education (>12 years).

^2^Living with one and/or two biological parents

^3^Women who reported being exposed to a specific type of violence, irrespective of whether they have been exposed to other types of violence.

**Table 2:** Associations between exposure to IPV during pregnancy, co-variables and postpartum depression.

Factor	EPDS≥10	EPDS<10	Bivariate analysis		Multivariate analysis	
			OR (95%CI)	p-value	AOR (95%CI)*∗*	p-value
*Emotional violence during pregnancy (n=1,274)*						
Yes	44 (10.7)	367 (89.3)	1.60 (1.07-2.41)	0.023	1.01 (0.60-1.69)	0.968
No	60 (7.0)	803 (93.0)	1		1	

*Physical violence during pregnancy (n=1,274)*						
Yes	13 (28.9)	32 (71.1)	5.08 (2.58-10.02)	0.001	2.75 (1.19-6.35)	0.018
No	91 (7.4)	1138 (92.6)	1		1	

*Sexual violence during pregnancy (n=1,274)*						
Yes	17 (13.6)	108 (86.4)	1.92 (1.10-3.35)	0.021	1.93 (1.01-3.73)	0.049
No	87 (7.6)	1062 (92.4)	1		1	

*Age of women* (years) *(n=1,274)*						
<25	53 (9.3)	520 (90.7)	1.30 (0.87-1.94)	0.201	1.92 (1.21-3.05)	0.006
≥25	51 (7.3)	650 (92.7)	1		1	

*Occupation of women (n=1,273)*						
Employed by government, private company or organization	43 (10.5)	365 (89.5)	2.25 (1.07-4.72)	0.032	4.21 (1.82-9.75)	0.001
Worker	20 (5.7)	329 (94.3)	1.16 (0.52-2.61)	0.716	1.28 (0.55-3.00)	0.569
Farmer	23 (13.9)	143 (86.1)	3.07 (1.38-6.85)	0.006	2.68 (1.13-6.35)	0.025
Unemployed/student	9 (5.3)	160 (94.7)	1.08 (0.42-2.78)	0.881	1.35 (0.47-3.81)	0.570
Small trade	9 (5.0)	172 (95.0)	1		1	

*Level of education* ${}^{\overset{\;}{\text{1}}}$ * (n=1,274)*						
Primary school	3 (12.5)	21 (87.5)	2.06 (0.59-7.26)	0.257	4.59 (1.04-20.29)	0.044
Secondary school	26 (11.4)	202 (88.6)	1.86 (1.09-3.16)	0.021	3.81 (1.92-7.56)	0.001
High school	39 (8.4)	426 (91.6)	1.32 (0.83-2.12)	0.241	2.35 (1.35-4.09)	0.002
University/college	36 (6.5)	521 (93.5)	1		1	

*Husband's preference for a specific sex of child* ${}^{\overset{\;}{\text{2}}}$ * (n=1,268)*						
Preference for son	56 (9.7)	519 (90.3)	1.77 (1.14-3.10)	0.056	1.98 (1.15-3.39)	0.014
Preference for girl	25 (9.3)	245 (90.7)	1.88 (0.99-3.20)	0.014	1.88 (0.99-3.60)	0.055
No preference	23 (5.4)	400 (94.6)	1		1	

*Gestational age at delivery* ${}^{\overset{\;}{\text{3}}}$ *(weeks) (n=1,257)*						
<37	10 (17.5)	47 (82.5)	2.56 (1.25-5.24)	0.01	2.33 (1.03-5.24)	0.042
≥37	92 (7.7)	1108 (92.3)	1		1	

*Family support after delivery* ${}^{\overset{\;}{\text{4}}}$ * (n=1,268)*						
Most of the time	38 (5.3)	681 (94.7)	0.77 (0.44-1.33)	0.341	0.84 (0.47-1.52)	0.565
Some of the time /rarely/never	45 (18.4)	200 (81.6)	3.08 (1.78-5.34)	0.001	3.46 (1.87-6.39)	0.001
All the time	21 (6.8)	288 (93.2)	1		1	

*∗* Adjusted for age of women, occupation of women, level of education, husband's preference for a specific sex of child, age of women at first pregnancy, mode of delivery, gestational age at delivery and family support after delivery

^1^Level of education was grouped into primary school (up to grade 5 years) and secondary school (grade 6-9 years) and high school (grade 10-12 years ) and higher education (>12 years).

^2^Husband's expressed preference of the sex of the unborn child (present pregnancy)

^3^Based on gestational age determined by ultrasound scanning performed during enrolment (3rd interview)

^4^Presence of at least one member of the family who has offered to take care of and support the mother and child.

## Data Availability

The data that support the findings of this study are available from the project, named “The Impact of Violence on Reproductive Health in Tanzania and Vietnam” (PAVE), Hanoi Medical University, but restrictions apply to the availability of these data, which were used under license for the current study and so are not publicly available. Data are however available from the authors upon reasonable request and with permission from the PAVE project.

## Abbreviations

ANC: Antenatal Care Clinics
AOR: Adjusted Odds Ratio
CHS: Commune Health Stations
CI: Confidence interval
DALYs: Disability-adjusted life years
EPDS: Edinburg Postnatal Depression Score
IPV: Intimate partner violence
OR: Odds Ratio
PPD: Postpartum depression
WHO: World Health Organization.
